# 

**Published:** 1877-02-20

**Authors:** 


☞ All communications relating to the business of The Record, for the year 1877, must be addressed to
DR. R. C. WORD,
Business Manager, South. Med. Rec.,
Atlanta, Ga.

☞ Brief and practical communications are solicited on all subjects pertaining to medicine, also reports of cases in practice.

☞ Send money by check, postal order, or registered letter.

☞ Write your name, post-office, county and State plainly.

☞ Our January number was gotten out under difficulties growing out of the unusual demand for press-work in the city, and the inability of the publishers to give the time and attention requisite to the proper execution of the work. The present issue will be found an improvement on the first, and the journal may be expected soon to attain to that neat and substantial form designed by the editors. The matter of the journal was highly practical and interesting, as evinced by testimonials already received. The present issue will also be found highly practical, and the editors will, in the future, labor still more assiduously to furnish our readers with more than a *quid pro quo* for the price of subscription.

